# Mounting Stroke Crisis in India: A Systematic Review

**DOI:** 10.7759/cureus.57058

**Published:** 2024-03-27

**Authors:** Vedant N Hedau, Tushar Patil

**Affiliations:** 1 Medicine, Jawaharlal Nehru Medical College, Datta Meghe Institute of Higher Education and Research, Wardha, IND; 2 Neurology, Jawaharlal Nehru Medical College, Datta Meghe Institute of Higher Education and Research, Wardha, IND

**Keywords:** low to middle income countries, disability-adjusted life years (dalys), stroke care challenges, india, stroke, cerebrovascular disorders

## Abstract

Stroke, a neurological disorder, has emerged as a formidable health challenge in India, with its incidence on the rise. Increased risk factors, which also correlate with economic prosperity, are linked to this rise, including hypertension, diabetes, obesity, sedentary lifestyle, and alcohol intake. Particularly worrisome is the impact on young adults, a pivotal segment of India's workforce. Stroke encompasses various clinical subtypes and cerebrovascular disorders (CVDs), contributing to its multifaceted nature. Globally, stroke's escalating burden is concerning, affecting developing nations. To combat this trend effectively and advance prevention and treatment strategies, comprehensive and robust data on stroke prevalence and impact are urgently required. In India, these encompass individuals with elevated BMIs, and those afflicted by hypertension, diabetes, or a familial history of stroke. Disparities in stroke incidence and prevalence manifest across India, with differences in urban and rural settings, gender-based variations, and regional disparities. Early detection, dietary changes, effective risk factor management, and equitable access to stroke care are required to address this issue. Government initiatives, like the National Programme for Prevention and Control of Cancer, Diabetes, Cardiovascular Diseases, and Stroke (NPCDCS) 2019, provide guidelines, but effective implementation and awareness campaigns are vital. Overcoming barriers to stroke care, especially in rural areas, calls for improved infrastructure, awareness campaigns, and support systems. Data standardization and comprehensive population studies are pivotal for informed public health policies.

## Introduction and background

Cerebrovascular disease (CVD) is a term used to describe all disorders that lead to stroke, which can be either ischemic or hemorrhagic. In low- and middle-income countries (LMICs), including India, the frequency of stroke increased by 100% between 1997 and 2008. This showed a 26% rise in stroke mortality worldwide during the previous 20 years [1]. Stroke continues to be the second most significant cause of death globally due to the increased mortality rate, according to the World Health Organization (WHO) 2020 [2]. Over the past four decades, there has been a statistically significant reduction in stroke incidence rates, with stroke incidence falling by 42% in high-income countries (HICs) and rising by more than 100% in LMICs [1]. Age, sex, low birth weight, ethnicity, and genetic variables are all irreversible risk factors for stroke [3]. A report of India's population-based stroke registries mentions that the majority of first-ever stroke cases reported hypertension (from Tirunelveli, 40.3%, to Cuttack, 75%), diabetes, and current tobacco use (from Varanasi, 19.3%, to Cuttack, 62.4%) [4]. Ischemic stroke (range 41.6% to 77.8%), hemorrhagic stroke (range 42.6% to 74%), and indeterminate stroke (range 2.0% to 35.9%) all had hypertension as their primary risk factor [4]. Young people's risk of stroke is considerably enhanced by smoking, drinking, having a higher BMI, having diabetes, and having high blood pressure [5,6]. A hospital-based multi-center prospective stroke registry in India to identify and recruit 10,000 acute stroke patients from 100 hospitals within the country conducted an interim analysis to determine aetiologies, clinical supervision, and outcomes with 5301 patients. According to the data, stroke patients had a number of highly hazardous factors, including heavy alcohol and cigarette use, diabetes, hypertension, and dyslipidemia [7].

The study found that stroke patients with higher frequencies of risk variables had higher short-term mortality [8]. Adopting coordinated care for stroke in LMICs is limited and insufficient, particularly in a nation like India, where the facilities available for rehabilitation are sparse [9]. The global burden of CVD has been rising, with stroke being the second most significant factor in fatalities worldwide, after ischemic heart disease in 1990 and the WHO 2020 factsheet [2,10]. A sizeable share of stroke deaths occur in LMICs, and these nations also experience more years of life lost to disability-adjusted life than high-income nations [11]. India has a greater cumulative incidence and crude prevalence of stroke than high-income nations [1]. This suggests that stroke is a significant health burden in India. Globally, there were around 25.7 million stroke survivors in 2013, along with 6.5 million stroke fatalities, 113 million years of life lost to disability, and 10.3 million new stroke cases [12]. Worldwide, the incidence of stroke is rising, mostly as a result of an older population and more risk factors such as type 2 diabetes and high blood pressure. Stroke occurrence among young people is increasing in LMICs [13]. There are regional, national, and ethnic differences in cardiovascular disease incidence, prevalence, and mortality. Due to their altered cardio-metabolic profiles and propensity for cardio-metabolic dysfunction, people from South Asian nations, especially India, have a disproportionately increased risk of cardiovascular disease [14]. To address the concerns of stroke prevention and treatment, reliable data on the burden of CVD in the Indian population is needed. To meet this demand, a comprehensive analysis of all community-based studies providing data on the mortality, prevalence, and incidence of stroke in rural, urban, and both population contexts is required. The present article aims to focus on the stroke crisis in India.

## Review

Search methodology

**Figure 1:**
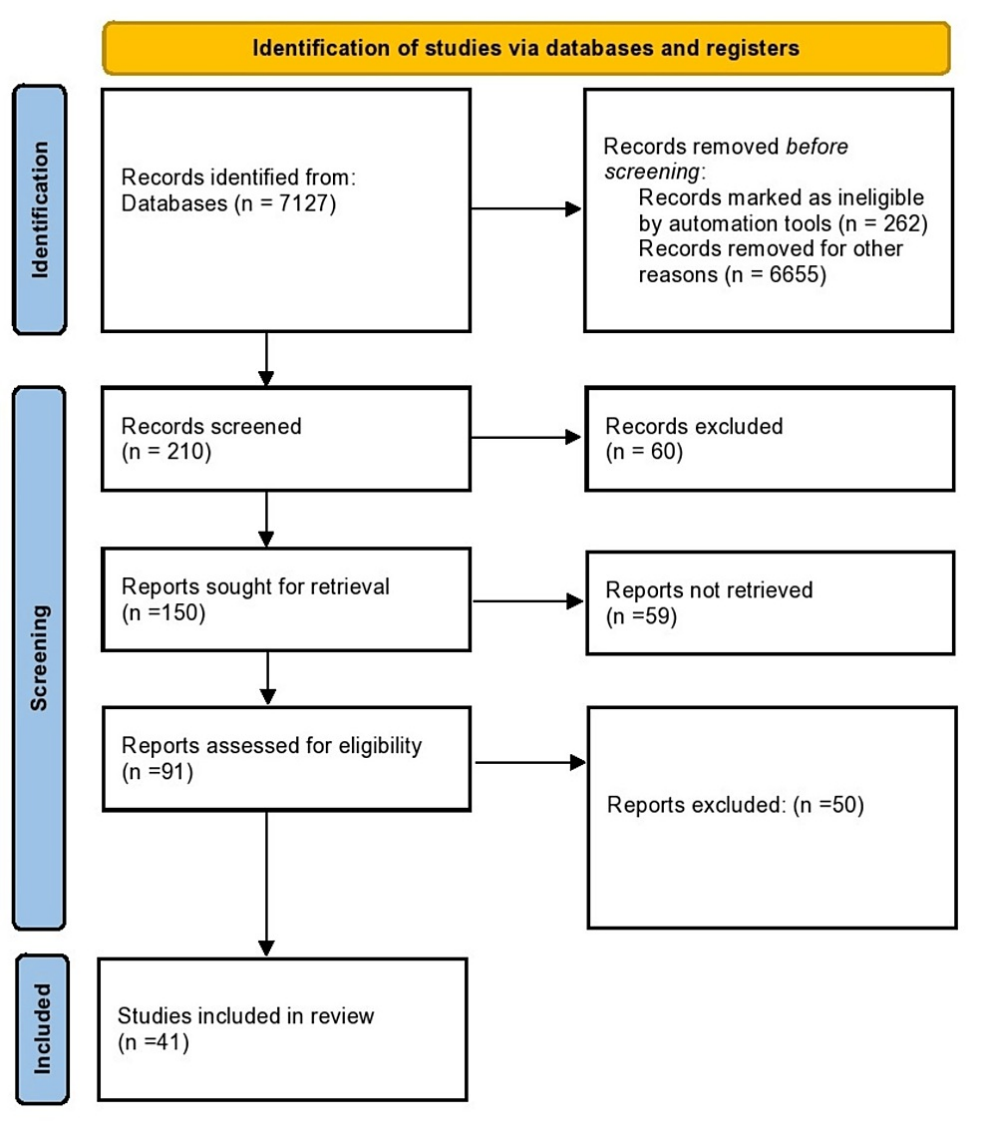
PRISMA flowchart of search strategy PRISMA: Preferred Reporting Items for Systematic Reviews and Meta-Analyses

A search was conducted on PubMed, CENTRAL, and other open-access sources using targeted keywords such as "stroke in India", "high-risk groups", "gender disparities," and "stroke incidence." The search focused on specific aspects, including high-risk groups, etiologic factors, detection and management, age-gender distribution, mortality, disability, rural-urban variations, and stroke care. Additionally, relevant references from the bibliographies of identified research were explored for comprehensive coverage. The review, anchored by Kamalakannan et al.'s (2017) foundational overview of stroke incidence and prevalence, integrated diverse perspectives [1]. The World Health Organization's fact sheet provided a global context and highlighted stroke's significance as a leading cause of death. Boehme et al. (2017) contributed insights into risk factors, genetics, and prevention, while NCDIR's (2021) report offered valuable Indian-specific data [3]. Lifestyle factors were addressed through studies by Mathur (2010) and Papathanasiou et al. (2015) [4,5]. Geographical diversity was enriched by insights from Burkina Faso (Dabilgou et al., 2020) and challenges in Zambia (Kayola et al., 2023) [8,9]. Global perspectives, including World Stroke Organization (2022), and expert insights from Lanas and Seron (2021) and Feigin et al. (2015) added depth to the discussion [11,12]. The research methodology involved a search strategy, utilizing precise keywords and MeSH terms. The inclusion of seminal studies, reports, and global perspectives ensured a comprehensive and well-rounded examination of stroke in India, spanning epidemiological, clinical, and public health dimensions. Figure 1 represents the search strategy of included studies in the review.

High-risk groups

Certain significant factors characterize high-risk groups for stroke in India. In contrast to HICs, LMICs, such as India, bear a much heavier burden of stroke. LMICs are responsible for more than 75% of worldwide fatalities caused by stroke and contribute to over 81% of disability-adjusted life years (DALYs) associated with stroke [15]. Within this context, it's evident that stroke disproportionately affects individuals in India, particularly those over the age of 50. The average body mass index (BMI) of stroke patients in India is 25.8 ± 4.3 kg/m2, considered in the overweight range, with 14.6% of patients classified as obese [16]. The fact that many of these persons have a history of diabetes, as well as elevated blood pressure, emphasizes the significance of these illnesses as stroke hazards in India. The overall risk is influenced by elements such as ischemic heart disease and a history of stroke in the family. Regional disparities exist in stroke incidence and prevalence rates across India, with some urban areas reporting comparatively lower annual stroke incidence rates (e.g., Rohtak and Southern Kolkata at 33 and 36 cases per 100,000 people, respectively) and others exhibiting substantially higher rates (e.g., Kolkata at 123.15 cases per 100,000 people) [17]. Notably, stroke incidence tends to be higher in women, often linked to uncontrolled hypertension. In rural West Bengal, the incidence rate is 123.57 cases per 100,000, and overall, these figures are greater than those of high-income nations, underscoring the substantial health burden posed by stroke in India [18]. Finding risk factors is essential because studies have linked a number of factors, including diabetes, hypertension, smoking, low hemoglobin levels, and elements of metabolic syndrome, as major causes of stroke [3,19]. The alarming increase in stroke incidence in the population in India is being addressed, and these data highlight the urgent need for targeted intervention and preventative initiatives. 

Age-gender dynamics

The average age of ischemic stroke casualties in India was 58.3 years admitted to hospitals between January 2012 and August 2014, all of India [20]. The study also reported that 67.2% of the patients were men [20]. It is believed that stroke patients in underdeveloped nations tend to be 15 years younger than individuals in advanced economies in terms of average age [21]. Age is a significant stroke risk factor, and India has a lower average age for stroke occurrence than Western nations. There was no difference in the average age at which strokes began between those in urban areas and those in rural areas. A young age group classified as 45 years or less experienced a stroke [22]. Nearly one-fifth of stroke patients in India who are admitted to hospitals are estimated to be under 40 years old [23]. Stroke can produce a significant burden economically as most of the population below 40 years is productive [21]. It affects the economy and families as most of the incidence occurs in males [24]. The data reveals variations in stroke incidence across different cities in India. Mumbai and Trivandrum have slightly higher mean ages for stroke patients (66 and 67 years, respectively), while Bangalore reports a lower mean age of 54.5 years for stroke patients. In Trivandrum, stroke rates per 1000 people per year are 7.1 for those aged 55 and above, rising to 13.3 for those aged 75 and above. Gender differences exist in stroke incidence; Men had a higher crude stroke incidence rate in Mumbai (149 per 100,000 person-years) than women (141 per 100,000 person-years), even after age adjustment. In Trivandrum, crude stroke incidence is higher in women (119 per 100,000 person-years) than men (115 per 100,000 person-years), but after age adjustment, men have a higher rate (143 per 100,000 person-years) than women (128 per 100,000 person-years). The Bangalore study shows a more significant occurrence of stroke in men (67% of cases), having a 2:1 male-to-female ratio [23]. These differences based on age and gender are statistically significant (P<0.01), indicating non-random variations in stroke rates [23]. Among ischemic stroke patients, 16.7% were between 18 and 45 years old, with the majority (83.4%) being male [25]. Most of the studies done are not comprehensive population studies. Most of these are limited to cities and areas. As the data constraints varied between cities, a comprehensive analysis of the population has not yet been conducted. The country is large, and each state has different food customs, cultural traditions, and lifestyles, making it difficult to identify risk factors.

Etiological factor 

India has a lower average age of stroke onset than Western nations, showing that aging is a substantial stroke risk factor [26]. In India diabetes, hypertension, the usage of tobacco, low hemoglobin, and certain heart conditions are identified as important risk factors for stroke [27]. High fasting glucose levels, high cholesterol, high triglycerides, and low HDL are causes of stroke risk, as is urbanization [27]. A major risk factor for stroke is identified as hypertension; hence, managing hypertension and quitting smoking are essential [4]. India has an excessive rate of smoking and chewing tobacco, which increases with lower socioeconomic position and education levels [28]. Women's health in India receives insufficient attention, leading to a high incidence and case fatality of stroke among older women, which is strongly correlated with the prevalence of hypertension [1,27]. The ages, sex, and regional distribution of the 2066 ischemic stroke patients with their baseline characteristics are represented in Table 1.

**Table 1:** Risk factors of stroke and scores on the National Institutes of Health Stroke Scale (NIHSS) [20] Table credit: Vedant N Hedau

Risk factors	Patient(%)
Hypertension	1257(60.8%)
Diabetes	737(35.7%)
Coronary artery disease	349(16.9%)
Hypercholesterolemia	298(14.4%)
Non-valvular atrial fibrillation/flutter	82(4.0%)
Rheumatic heart disease	115(5.6%)
Prior transient ischemic attack	159(7.7%)
Prior ischemic/hemorrhagic stroke	410(19.8%)

Admission scores on the National Institutes of Health Stroke Scale (NIHSS), with a median of 10. Risk factors are common, with hypertension making up 60.8% of cases, diabetes mellitus accounting for 35.7%, cigarette smoking accounting for 32.2%, and atrial fibrillation making up 4%. Large arterial strokes (29.9%), cardiac strokes (24.9%), and small artery strokes (14.2% of all strokes) are the most common etiologic subtypes. 81% of patients undergo brain imaging, and all patients receive computed tomography or magnetic resonance imaging [20]. 

Stroke detection, management, and prevention in India

**Figure 2:**
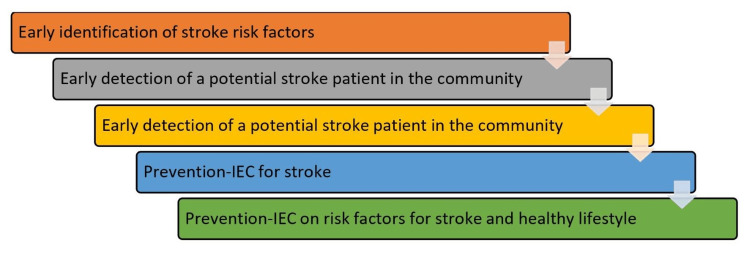
A framework of stroke management by the National Programme for Prevention and Control of Cancer, Diabetes, Cardiovascular Diseases and Stroke (NPCDCS) [31] IEC: Information, education, and communication Figure credit: Vedant N Hedau

The guidelines for managing and averting stroke are offered by the National Programme for Prevention and Control of Cancer, Diabetes, Cardiovascular Diseases and Stroke (NPCDCS) 2019 [29]. These guidelines mention all the details about stroke management detection, prevention, and management of stroke. They State the frame and system created for the management of stroke. With the requirement of health personnel and technical facilities essential for stroke diagnosis. India's rural population (% of the total population) was reported to be around 65% in 2022 [30]. In stroke care, an accredited social health activist (ASHA) plays a pivotal role in early detection by conducting assessments and raising community awareness. ASHA refers high-risk individuals to appropriate facilities and provides ongoing support. The auxiliary nurse midwife (ANM) complements this effort by aiding in risk assessment, referral, and follow-up care. The primary healthcare center (PHC) team, including the medical officer, lady health visitor, and laboratory technician, ensures diagnostic confirmation and treatment initiation, as well as managing complications, as shown in Figure 2 [31].

Additionally, all stakeholders emphasize the urgency of stroke referrals. Together, this collaborative network works to detect risk factors, raise awareness, provide education, and ensure timely care, enhancing stroke care outcomes at the community level. The lack of availability of advanced services and trained specialists for managing stroke in rural India. Most of the population has to come to districts to seek advanced care and diagnostics services. As we know, in stroke cases, time plays a crucial role. However, due to a lack of awareness of the severity of the situation, patient care is often delayed, leading to disability and mortality. 

Stroke mortality and disability-adjusted life years (DALYs) in India

Stroke is a serious public health issue in India, and it has a big global impact. India is responsible for roughly 13.3% of the worldwide DALYs lost as a result of stroke [32]. According to estimates from the Global Burden of Disease (GBD) research, stroke was the sixth most common cause of DALYs lost in the nation in 2017 [33]. The GBD study also found significant regional inequalities, with West Bengal, Assam, Odisha, and Chhattisgarh having the highest rates of stroke. States like Mizoram, Sikkim, and Delhi, on the other hand, revealed substantially lower incidences of stroke-related DALYs [34]. Fatality rates vary across rural and urban regions [17]. These findings underscore the pressing need for targeted interventions and healthcare strategies to address stroke prevention and management, particularly in regions with the highest burden. Recognizing these disparities is crucial in developing effective Public health measures designed to lessen the overall impact of stroke throughout India.

Stroke care's challenges and shortcomings

In India, despite government initiatives and strategic planning for stroke detection, prevention, and management, there are significant challenges on the ground. The country's transition phase of economic development, with a majority residing in rural areas, lacks adequate stroke care facilities [35]. This rural-urban divide is exacerbated by the limited access to education and healthcare in LMICs, making it challenging for people to recognize stroke symptoms and risk factors. Resource limitations often lead to delayed or insufficient preventive efforts and highlight healthcare disparities, particularly in LMICs [36]. Specialized stroke centers, telemedicine, and advanced treatments are often inaccessible, causing delays in care. Transportation and infrastructure constraints further hinder timely access to healthcare facilities. Moreover, post-stroke rehabilitation and long-term care services are frequently inadequate and financially burdensome for patients and their families [37]. Addressing these challenges in LMICs necessitates improvements in healthcare infrastructure, cost-effective interventions, community-based education, and international collaboration to bridge the disparities in stroke care worldwide [36]. These efforts should be catered to the specific needs of LMICs to ensure equitable access to stroke prevention, treatment, and support. The issue is made worse by the deficit of comprehension of the seriousness of risk factors like hypertension, addiction to cigarettes, and diabetes, as well as the low compliance even among cases that have been diagnosed. Additionally, Western parameters for predicting stroke may not accurately apply to the South Asian population [38]. Despite government financing schemes, access to complete treatment is further hampered in rural regions by economic restraints [39]. Raising awareness and addressing these gaps is vital to enhancing stroke care in India. Table 2 depicts the characteristics of the included studies in the article.

**Table 2:** Characteristics of the study included in the article DALYs: Disability-adjusted life years; GBD: Global Burden of Disease; HALE: Health-adjusted life years; LMICs: Low- and middle-income countries; NIHSS: National Institutes of Health Stroke Scale; NPCDCS: National Programme for Prevention and Control of Cancer, Diabetes, Cardiovascular Diseases and Stroke; SDI: Socioeconomic Deprivation Index

Author/organization	Year	Findings
Kamalakannan S, et al. [1]	2017	Research on stroke in India highlights the necessity of a concerted national and state-level effort to examine the incidence of stroke in the country.
World Health Organization (WHO) [2]	2019	According to the overall number of lives lost, the top three global causes of death are related to three main areas: stroke is listed second.
Boehme AK, et al. [3]	2017	This suggests that because of particular stroke processes and other risk factors, both frequent and unusual genetic variants may affect the risk of stroke from more common causes; genetic elements, especially those that interact with the environment.
National Centre for Disease Informatics and Research (NCDIR) [4]	2021	Stroke research was conducted in five cities throughout various regions of India. Based on the first stroke ever recorded in terms of risk factors, age, gender, disability, and death.
Mathur RK [5]	2010	Diabetes, hypertension, and cigarette smoking are risk factors that contribute to the advancement of atherosclerosis and stroke.
Papathanasiou G, et al. [6]	2015	Explains how, in healthy young adults, high blood pressure is related to body mass index, smoking, and physical activity.
Taylor FC, et al. [7]	2012	India is experiencing an epidemic of strokes, which calls for immediate action in the shape of a stroke policy that is tailored to the nation's requirements and based on data.
Dabilgou AA, et al. [8]	2020	According to the study, stroke patients had greater short-term mortality rates when their risk indicators were more frequently present.
Kayola G, et al. [9]	2023	Examines potential tactics that might enhance the availability and caliber of rehabilitation services in low- and middle-income countries (LMICs), such as the establishment of inpatient stroke units, more chances for rehabilitation specialists to receive training, delegating tasks to available caregivers or healthcare professionals, telerehabilitation, and community-based rehabilitation programs.
World Stroke Organization (WSO) [10]	2022	If one were to measure the world's top causes of death and disability together using disability-adjusted life years (DALYs) lost, stroke would still rank second globally.
Lanas F, et al. [11]	2021	Stronger preventive measures should result from the higher incidence of ischemic stroke as compared to Chile's Global Burden of Disease (GBD) estimate. The study demonstrated that if scientifically sound procedures are followed, high-quality epidemiological research may be conducted in environments with minimal resources.
Feigin VL, et al. [12]	2013	A divergence trend was observed between industrialized and developing nations, with a notable rise in DALYs and mortality in the former and no discernible shift in the proportionate share of DALYs and stroke deaths in the latter.
Gorthi S, et al. [13]	2022	Concentrate on the long-term results of young stroke patients in Ludhiana in order to better comprehend and mitigate the numerous aftereffects of stroke that extend beyond its immediate aftermath.
Pursnani S, et al. [14]	2020	In the context of a higher load of conventional cardiovascular disease risk factors, South Asian ethnicity has been linked to an elevated risk of cardiovascular disease.
Pandian JD, et al. [15]	2020	Compared to high-income nations, the burden of stroke is greater and growing in LMICs. There are global guidelines and strategies for stroke care implementation, but establishing stroke services in LMICs is fraught with difficulties.
Ram CV, et al. [16]	2021	Diabetes and hypertension are the two main risk factors for stroke in India and should be managed similarly to other high-risk populations around the world in order to prevent strokes. The correlation between risk factors and the severity of a stroke is demonstrated by the National Institutes of Health Stroke Scale (NIHSS) scores.
Khurana S, et al. [17]	2021	According to a systematic study and meta-analysis, India has a very high stroke burden.
Bhattacharya S, et al. [18]	2005	This study revealed that smoking, heart disease, and hypertension are significant risk factors for stroke, with India having a higher age-adjusted incidence rate of the condition than developed nations.
Chen R, et al. [19]	2016	Diabetes, particularly ischemic strokes, is a significant modifiable risk factor for stroke. In both ischemic and hemorrhagic strokes, hyperglycemia during the initial period is linked to unfavorable outcomes.
Sylaja PN, et al. [20]	2018	The advancement of stroke care in India and the improvement of stroke guidelines would benefit from the utilization of extensive and innovative clinical imaging data.
Tripathi M, et al. [21]	2011	Further research may reveal that stroke rates among young Indians are not significantly different from those in other nations, but there are still a lot of ramifications for emerging nations. Preventive actions could significantly save expenses and ease the family's emotional strain.
Griffiths D, et al. [22]	2011	Women appear to have a higher incidence of stroke than males under the age of 30, and throughout the puerperium, women are more likely to experience hemorrhage and infarction. For young women, further medical history is crucial, including oral contraceptive pill use and antiphospholipid antibody tests.
Pandian JD, et al. [23]	2013	The government and corporate sectors must work together in a concerted effort to address India's growing stroke epidemic.
Donkor ES [24]	2018	Improving the effectiveness of stroke treatment may require an understanding of the infection patterns in stroke patients, as well as the antibiotic susceptibility patterns of the bacterial etiological agents.
Dash D, et al. [25]	2014	In North India, 16.7% of stroke patients are young adults. There are many patients classified as having unknown or other determined causes, and risk factors are rather common. The findings emphasize the necessity of comprehensive patient work-ups and vigorous therapy of conventional risk factors to determine the etiology of stroke in India.
Yousufuddin M, et al. [26]	2019	With the overall incidence and mortality linked to stroke, it is critical to maintain attention on comorbidity in addition to risk variables.
Das S, et al. [27]	2016	Further interventional studies are needed in India to determine the effectiveness of preventive medications such as antiplatelets and antihypertensives, as well as to investigate the attitudes and levels of awareness across different ethnic groups.
Thakur JS, et al. [28]	2018	In addition to variations in the kind, quantity, and dependence of tobacco use, there are disparities in the prevalence of tobacco use across higher and lower socioeconomic categories, which exacerbates the variations in the illness burden caused by tobacco use.
NPCDCS [29]	2019	Sharp increases in the prevalence of stroke are being caused by changes in lifestyles, behavioral patterns, demographics (aged population), sociocultural factors, and technological improvements.
Economic survey highlights thrust on rural development [30]	2023	According to the survey, 47% of the nation's population depends on agriculture for a living, while 65% of people live in rural areas as of 2021 estimates.
NPCDCS [31]	2019	This guideline was created with healthcare professionals managing stroke victims in mind. The goal is to support them in making optimal decisions for each patient utilizing the available evidence at the primary and secondary levels of the healthcare delivery system. The more frequent clinical queries that arise in regular practice are the main focus.
Sudharsanan N, et al. [32]	2019	These findings highlight the critical need for enhanced stroke prevention and treatment services in rural India as well as increased surveillance.
Kyu HH, et al. [33]	2017	The findings indicated that throughout the previous 28 years, there have been significant advances in health around the world. However, there are significant differences in disease burden, years lived in poor health, and health-adjusted life years (HALE) between sexes as well as among socioeconomic deprivation index (SDI) quintiles and countries.
Dandona L, et al. [34]	2017	In India in 2016, ischemic heart disease, chronic obstructive pulmonary disease, diarrheal illnesses, lower respiratory infections, and cerebrovascular disease were the top five individual causes of DALYs.
Sridharan SE, et al. [35]	2009	When it comes to the epidemiology of stroke, industrialized and developing nations share more characteristics than not. Rural stroke patients are less likely to receive the best possible diagnosis and care than their urban counterparts.
Yan LL, et al. [36]	2016	In LMICs, leading a healthy lifestyle that includes quitting smoking, eating a balanced diet, exercising, and controlling one's weight are crucial tactics for primary and secondary stroke prevention.
Kamalakannan S, et al. [37]	2016	Creating a patient-centered, creative, accessible, and culturally appropriate rehabilitation intervention is crucial for public health given the paucity of rehabilitation resources in India.
Gunarathne A, et al. [38]	2009	The review highlights the need for focused healthcare measures and the increased burden of cardiovascular disease among South Asians, which makes more studies on ischemic stroke crucial.
Mathur S, et al, [39]	2019	Patients frequently live far away from facilities for specialized care. Many rural hospitals have understaffed their specialized departments, cut back on services, and tightened budgets, which has left a large number of patients underserved.
Smajlović D [40]	2015	By comparing incidence rates across nations and examining trends over time, these researches will shed light on the underlying etiologic mechanisms and bring clarity to the situation. The main approach to lowering the morbidity and mortality associated with juvenile stroke is prevention; however, no particular guidelines or recommendations exist.
Kruk ME, et al. [41]	2018	Everyone needs to be able to rely on getting excellent care that will both enhance their health and win their trust. It's time to reconsider our previous strategies and make more investments in and demands of this important health determinant.

Discussion

The increasing Indian stroke prevalence is a pressing concern, primarily driven by risk factors like obesity, diabetes, alcoholism, hypertension, and sedentary lifestyles. These variables, which are made worse by economic expansion, call for immediate preventive steps and lifestyle adjustments. Due to a lower mean age of onset than in Western nations, young adults (a significant portion of the workforce) are particularly affected by stroke, which creates economic and cultural problems [40]. It is critical to address these risk factors and educate younger people about them.

Stroke care disparities between rural and urban areas pose serious problems because early diagnosis and treatment are hampered by poor access to healthcare and education in rural India. These gaps must be filled, and underserved areas' healthcare infrastructure must be improved. Although government programs like the NPCDCS 2019 offer recommendations, their effectiveness depends on their efficient implementation and awareness-raising efforts [29].

Stroke data collecting needs to be standardized, and there should be more thorough demographic studies, especially in rural regions because there aren't enough reliable and comprehensive data on the disease [41]. An understanding of the numerous co-morbidities and risk factors, such as smoking, diabetes, elevated blood pressure, and low hemoglobin levels, emphasizes the necessity of early detection and lifestyle changes [3]. For equitable care, it is essential to address gender differences in the frequency of stroke.

## Conclusions

The escalating frequency of stroke in India poses a critical health concern, closely tied to the rising occurrence of risk factors, such as obesity, diabetes, alcoholism, hypertension, and sedentary lifestyles. This health challenge takes an alarming toll on young adults, a pivotal segment of India's workforce, who experience strokes at a significantly younger age compared to their Western counterparts, leading to significant mortality and disability, which affects families and the country. To confront this complex issue, several crucial medical insights must be underscored. Prevention emerges as the linchpin of stroke management, necessitating the identification of high-risk groups, including individuals over 50, those with elevated BMI, and those afflicted by hypertension, diabetes, or familial history of stroke. Strong preventive interventions that include lifestyle changes, early detection, and the successful management of these underlying hazards should be the main focus. Moreover, addressing regional disparities is paramount as urban-rural divides and gender-based variations persist. In order to close these disparities, it is crucial to implement organized awareness efforts, community-based education, and fair access to stroke healthcare services. The scarcity of comprehensive stroke-related data underscores the necessity for standardized data collection and extensive population studies, especially in rural areas, to inform evidence-based public health policies accurately.

Furthermore, the identified risk factors in India, such as hypertension, diabetes, tobacco use, and low hemoglobin levels, emphasize the critical importance of early detection and effective management. Healthcare providers must prioritize screening, education, and intervention in managing these risk factors. Gender disparities in stroke incidence are evident, demanding a focused approach and gender-specific strategies to provide equitable stroke care. Overcoming barriers to stroke care, notably limited access to advanced services in rural areas, delayed care due to low awareness, and the significant financial burdens borne by patients and their families, necessitates comprehensive solutions. Enhancing healthcare infrastructure post-stroke rehabilitation services, raising awareness through targeted campaigns, and establishing robust support systems are essential to surmounting these challenges. In a holistic sense, the effective management of the mounting stroke challenge in India calls for a multifaceted approach that encompasses prevention, research, equitable access to care, and the dissemination of awareness both among the general public and healthcare providers. India may make tremendous progress in reducing the effects of stroke and enhancing the general health of its population by embracing these important medical concepts.
